# Advances in In Vitro and In Vivo Bioreactor-Based Bone Generation for Craniofacial Tissue Engineering

**DOI:** 10.34133/bmef.0004

**Published:** 2023-01-31

**Authors:** Emma Watson, Antonios G. Mikos

**Affiliations:** Department of Bioengineering, Rice University, Houston, TX 77030, USA.

## Abstract

Craniofacial reconstruction requires robust bone of specified geometry for the repair to be both functional and aesthetic. While native bone from elsewhere in the body can be harvested, shaped, and implanted within a defect, using either an in vitro or in vivo bioreactors eliminates donor site morbidity while increasing the customizability of the generated tissue. In vitro bioreactors utilize cells harvested from the patient, a scaffold, and a device to increase mass transfer of nutrients, oxygen, and waste, allowing for generation of larger viable tissues. In vivo bioreactors utilize the patient’s own body as a source of cells and of nutrient transfer and involve the implantation of a scaffold with or without growth factors adjacent to vasculature, followed by the eventual transfer of vascularized, mineralized tissue to the defect site. Several different models of in vitro bioreactors exist, and several different implantation sites have been successfully utilized for in vivo tissue generation and defect repair in humans. In this review, we discuss the specifics of each bioreactor strategy, as well as the advantages and disadvantages of each and the future directions for the engineering of bony tissues for craniofacial defect repair.

## Introduction

Large-volume bone loss in the craniofacial region, whether due to trauma, congenital defects, infection, or cancer, requires a complex and well-designed repair strategy. The face is an important identifier of self and plays a critical role in nonverbal communication. Many tissue types exist in close proximity, with the color and texture of the overlying skin, the bulk and shape of the muscle and fat, and the structure of the underlying bone all contributing to the appearance of the patient and their happiness with the reconstruction. However, a robust underlying bony skeleton is necessary for both form and functioning, with masticating and talking subjecting the mandible to substantial mechanical forces, requiring use of robust tissues, plates, and screws for efficacious repair. Balancing the need for aesthetics and function can be difficult when only native tissues are available for reconstruction. In the United States, the most commonly used vascularized flap for the mandible is the fibular flap, but scapular or iliac crest flaps are also utilized [1]. Although fibular flap harvests can sometimes lead to donor site morbidity (such as foot drop or pain and sensory changes), these long, essentially straight bones have been used successfully in reconstruction to reapproximate mandibular geometry. Ribs have also been utilized in zygomatic arch reconstruction due to similarities in contour, but the mismatch in cortical thickness has been an issue [2]. Smaller bones, such as the nasal bone or orbital floor, can be reconstructed by carefully piecing together grafts harvested from the calvarium [3]. When the calvarium is harvested as bicortical grafts, the dura and underlying vascular sinuses are at risk of damage during the surgery, but several pieces of full-thickness outer cortex graft are useful for reconstructing the acute angles of the nasal and periorbital areas.

With the advent of virtual surgical planning (VSP) and 3D printing, the use of contouring, cutting guides, and pre-bent plates has further increased the fidelity of the reconstructions. VSP involves the use of imaging and computer-aided design to perform virtual surgery before the operating room or to create 3D printed models of the patient-specific anatomy, allowing the surgeon to pre-bend plates and further visualize the patient’s anatomy in 3 dimensions (3D) [4]. Additionally, several companies that provide surgical plates and screws can create 3D printed cutting guides and titanium plates, leading to increased fidelity of repair. Even though bioengineering has improved these native flap strategies in recent years, they still use another bone with different initial structure and purpose to reapproximate craniofacial geometry and function, relying heavily on surgeon skill and the hardware/guides available for the contouring procedure. Generating bone tissues of customized geometries without sacrificing robust mechanical properties would alleviate concerns with donor site morbidity, facial contour, and function. In this review, we discuss recent trends in tissue engineering for creating customized tissues for craniofacial reconstruction.

## Bone Tissue Engineering

Tissue engineering as a field was officially coined in 1988 with the goal of restoring, maintaining, or improving tissue function. Today, the tissue engineering tetrad involves cells, signaling molecules, mechanical forces, and scaffolds [5]. Not only must each of these factors be considered independently, the interplay between these factors is also important. A scaffold with the perfect architecture will not be effective without considering the biocompatibility of the material or the ability for cells to adhere to and remodel the construct. Likewise, the correct cells with the incorrect signals will not result in successful tissue engineering.

**Fig. 1.**
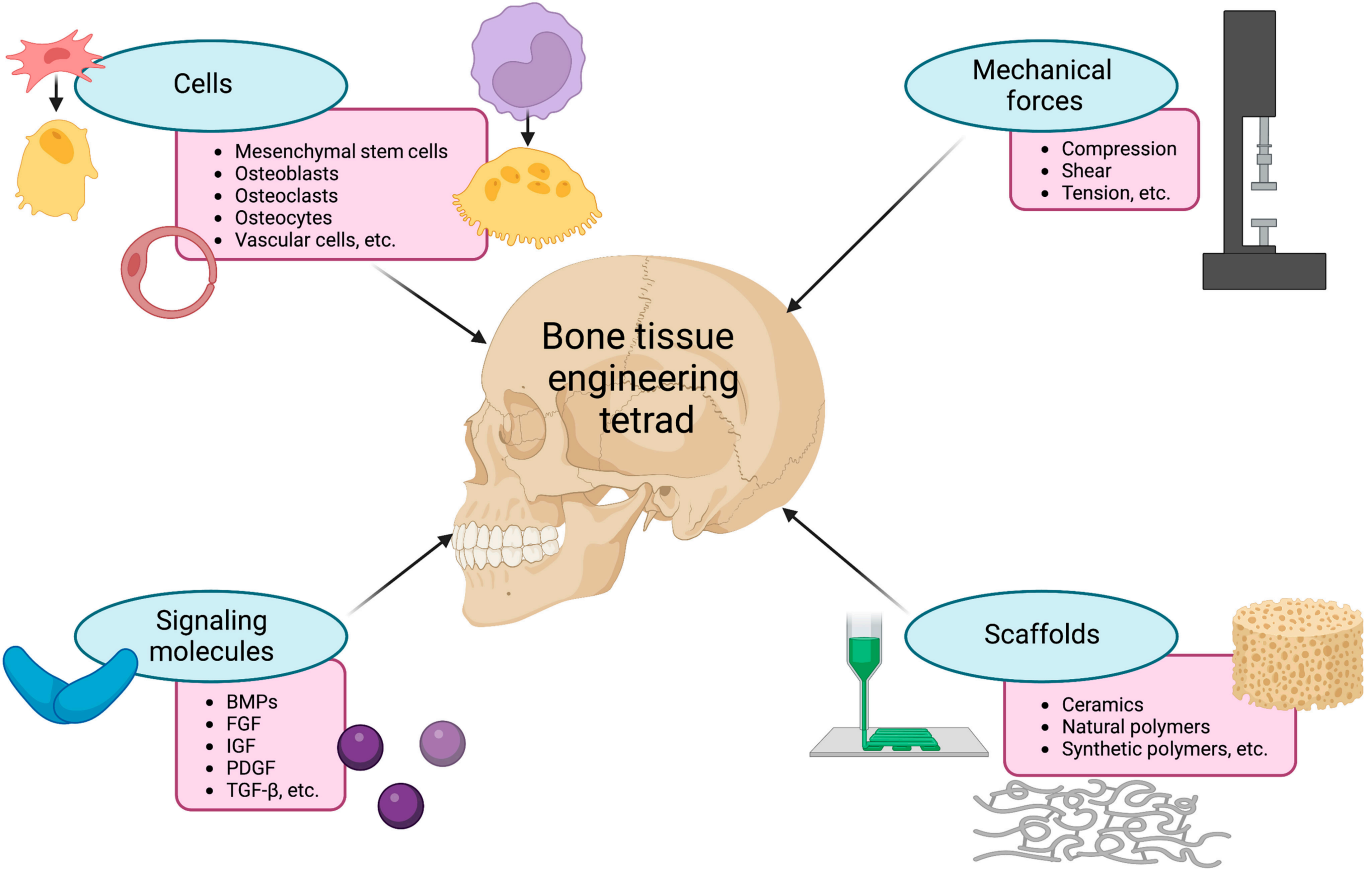
The bone tissue engineering tetrad depicting some of the key cells, growth factors, scaffold materials, and mechanical forces utilized to successfully generate bone.

The tissue engineering paradigm for bone is given in Fig. 1. Osteoblasts are the major cells responsible laying down extracellular matrix (ECM) and mineralizing the bone. They differentiate from mesenchymal stem cells (MSCs) upon production of transcription factors like Runx2 and Osterix [6]. Bone undergoes near-constant remodeling, with the osteoblasts forming new bone as the osteoclasts degrade it. The osteoclasts differentiate from macrophage/monocyte hematopoietic stem cells after activation of nuclear factor κB (RANK) signaling [7]. A complex interplay exists between the osteoblasts and osteoclasts, due in part to signals released from the osteocytes. The osteocytes are terminally differentiated osteoblasts that have become encapsulated within mineralized bone, releasing molecules such as osteoprotegerin (OPG) and RANK-ligand (RANK-L) [8]. To generate robust bone, it is important to include these cells, either in their differentiated forms or as stem cells with the appropriate signals to encourage differentiation.

Bone is a complex mixture of organic and inorganic compounds. The organic components (40%) are composed primarily of collagen type I, and the inorganic (60%) of hydroxyapatite [9]. The scaffolds utilized in tissue engineering are not necessarily a direct copy of this composition, with many biocompatible, biodegradable compounds being utilized. The biocompatibility is critical for any scaffold that will be in contact with cells, while the biodegradability allows for the cells to degrade, deposit, and remodel their own ECM using the initial scaffold as a guideline. Bone tissue has been successfully engineered on scaffolds made of collagen [10], poly(ε-caprolactone) [11], and β-tricalcium phosphate (β-TCP) [12], among others. Just as a variety of materials have successfully been utilized to generate bone in vitro, many different manufacturing methods can be utilized to create the scaffolds. Scaffolds can be generated as complex 3D structures mimicking the trabecular and cortical regions of bone [13], or as more simple structures, like films [14], coatings [15], or particles [16]. Several commercially available and clinically utilized acellular scaffold products exist for bone tissue engineering, such as MasterGraft (biphasic calcium phosphate: 85% hydroxyapatite, 15% β-TCP), Bio-Oss (bovine bone with trabecular architecture after removal of organic components), and DBX (demineralized bovine bone with trabecular architecture).

Several growth factors play an important role in the formation of bone and have been leveraged with success for bone tissue engineering. Bone morphogenetic proteins (BMPs) are members of the transforming growth factor-β (TGF-β) superfamily and cause increases in transcription factors (such as Runx2) after binding to a seronine–threonine kinase receptor and signal transduction via Smad [17]. BMPs are osteoinductive and have been released from sponges, 3D-printed scaffolds, hydrogels, and microparticles composed of natural polymers, synthetic polymers, or ceramics [18]. The Food and Drug Administration (FDA) has approved INFUSE (rhBMP-2, initially for only spinal fusion with expanded applications to long bones and craniofacial skeleton repair since) [19] and OP-1 (rhBMP-7, no longer on the market but initially approved via a Humanitarian Device Exemption) [20]. Platelet-derived growth factor (PDGF), specifically isoform PDGF-BB, can increase osteogenic differentiation via the phosphatidylinositol 3 kinase and extracellular signal-related kinase 1/2 signaling pathways [21]. Several commercially available products that leverage rhPDGF-BB for bone regeneration include Augment (hindfoot and ankle arthrodesis) and GEM 21S (periodontal defects). Additional growth factors utilized in bone tissue engineering include fibroblast growth factors (FGFs), insulin-like growth factors (IGFs), and vascular endothelial growth factor (VEGF). Recent advances have led to the development of small molecules that also encourage osteoblastic differentiation, such as Peptide-15, which is now available commercially as iFactor [20]. Furthermore, “osteogenic media” is frequently utilized to encourage the differentiation of MSCs in vitro and does not contain any of the traditional growth factors [22]. This cocktail consists of the original media, ascorbic acid, dexamethasone, and sodium β-glycerophosphate, with or without fetal bovine serum.

Mechanical forces also play a role in the development of bone. While not crucial—MSCs in osteogenic media will differentiate without the need of additional mechanical stimuli—compression [23], tension [24,25], and shear stresses [26] have all been shown to increase osteogenic differentiation. Various pathways have been proposed for the role of mechanical stimuli, several involving epigenetics and the expression of sonic hedgehog [27] and notch [28]. Additionally, lack of mechanical stimulation and other signaling has shown that MSCs are more likely to undergo adipogenic differentiation [29]. The addition of compression, tension, or shear stresses can encourage osteogenic differentiation and should be considered when planning a tissue engineering strategy for the generation of bone.

Given the understanding of the cells that deposit and remodel bone matrix, the knowledge of important growth factors and development of other osteogenic small molecules, and the ever-increasing manufacturing methods of scaffolds, one may wonder what is preventing the creation of customized bone constructs for every patient who needs one. The limitations affecting bone tissue engineering are similar to those impacting the engineering of other tissues—nutrient and oxygen delivery. In the human body, most cells (save for in avascular tissues like articular cartilage) exist within 200 μm of capillaries [30]. In bone, the Haversian and Volkmann canals are separated between 100 and 300 μm, placing most bone cells within 150 μm of the supply of nutrients, oxygen, and signals as well as crucial removal of waste [31]. Generation of bone tissue-engineered constructs of several cubic centimeters requires careful consideration of how nutrient transfer and waste removal will occur for the cells at the heart of the construct. Recent approaches to overcoming this limitation have involved 3D-printing vascular channels within the scaffolds [32], spatial patterning of bone and endothelial precursors [31], and bioreactors.

## Bioreactors

Bioreactors can be generally defined as devices that use mechanical means to influence biological processes [33]. With their basis in chemical reactor engineering, spinner-flask, rotating-wall, hollow-fiber, and direct-perfusion bioreactors have all been utilized to successfully culture cellular constructs [34]. In addition to eliminating some of the concerns with diffusion limitations due to the circulation of the culture media, these devices allow for the control of mechanical or shear forces applied to cells, affecting their differentiation. In addition, these in vitro bioreactors allow for a tightly controlled and reproducible environment in which to generate tissues or study pathological processes. As it is difficult to fully recapitulate the physiological environment in vitro, in vivo bioreactors have more recently become of interest to researchers.

In vivo bioreactors have been developed and can be defined as bioreactors implanted within the body to make use of the body’s natural regenerative capacity to create tissues [35]. Although the properties of what is initially implanted can be tightly controlled, the further growth and development of the tissue is heavily reliant on the individual—and more specifically the site—into which the bioreactor is implanted. Bone tissue has been successfully generated within both in vitro and in vivo bioreactors.

## In Vitro Bioreactors

**Fig. 2.**
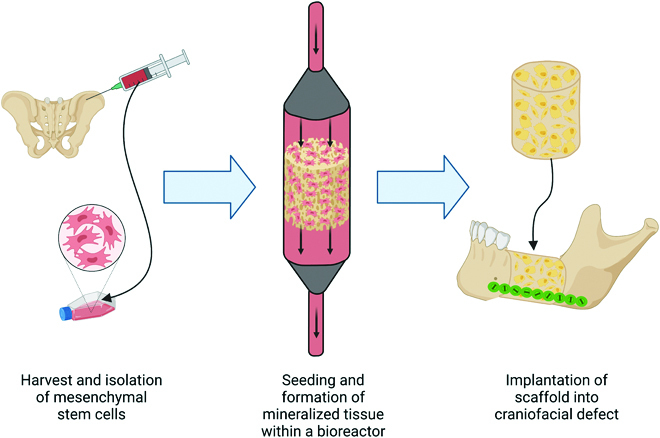
The strategy for in vitro bioreactor-based bone generation. Bone marrow is harvested from a bone such as the iliac crest, and mesenchymal stem cells (MSCs) are isolated and expanded. The MSCs are seeded on a scaffold and cultured in an in vitro bioreactor. After several weeks, the MSCs have differentiated into osteoblasts and begun to deposit new mineralized matrix. The engineered bone can then be transferred to the defect site.

With the goal to replace a bone within the craniofacial skeleton, in vitro bioreactors could be used to initiate bone formation before implantation in the defect site. As shown in Fig. 2, cells are harvested for the person in need of the bone replacement. Potential sources for cells to culture within the bioreactor are bone marrow- or adipose-derived MSCs [36]. A scaffold is formed from any of the materials discussed above, and utilizing novel fabrication methods (such as 3D printing), a customized scaffold is developed. The harvested cells and generated scaffold are then cultured within the bioreactor. Growth factors, osteogenic media, or osteoinductive compounds present within the scaffold or media may be used to further encourage osteogenic differentiation. After several weeks in culture, the scaffold can be harvested, and a surgery can be performed to implant the cell-laden mineralized bony scaffold. A variety of different in vitro bioreactor systems have been explored as potential methods for engineering bone tissue for scaffolds of a variety of sizes, materials, and porosities. The sections below will further discuss these in vitro bioreactors and will focus on scaffolds that are at least 0.5 cm^3^ in volume and may be of use in the repair of smaller nasal or larger mandible defects (Table 1).

**Table 1.** In vitro bioreactor studies that generate tissues of >0.5 cm^3^. Results relative to the control, most commonly static culture.

	Scaffold	Cell type	Parameters	Results	Animal model	Source
Spinner-flask	PLGA, 12.7 mm diameter, 6 mm height	Rat bmMSCs	30 rpm, 120 ml, 21 days	↑ ALP, ↑ osteocalcin, ↑ calcium	No	[39]
	Silk, 15 mm diameter, 5 mm height	Human bmMSCs	50 rpm, 100 ml 84 days	↑ proliferation, ↑ differentiation early;↑ calcium, ↑ compressive modulus late	No	[40]
Rotating-wall	PLGA, 12.7 mm diameter, 6 mm height	Rat bmMSCs	6 foams 20 rpm, 14 days	More homogeneous seeding, — proliferation	No	[44]
	PLGA, 12.7 mm diameter, 6 mm height	Rat bmMSCs	6 foams, 30 rpm, 21 days	— proliferation, ↓ calcium, ↓ALP	No	[39]
Perfusion-based	PLGA, 12.7 mm diameter, 6 mm height	Rat bmMSCs	Direct, 1.8 ml/min, 2 weeks	↑ proliferation, enter ↑ ALP/cell	No	[44]
	Decellularized bovine bone, TMJ geometry	Human bmMSCs	Direct 1.8 ml/min, 4 weeks	↑ proliferation, enter ↑ mineralization	No	[49]
	β-TCP, 12 mm diameter, 6 mm height	Human bmMSCs	Indirect, 1.5 ml/h, 3 weeks	↑ proliferation, enter— ALP/cell	No	[51]
	Polyurethane, 24 mm diameter, 6 mm height	MC3T3-E1	Direct, 1.0 ml/min, 8 days	More homogeneous seeding, ↑ metabolic activity	No	[52]
	β-TCP, 14 mm diameter, 33 mm height	Human MG63	Direct, 3 ml/min, 4 weeks	↑ proliferation, enter ↑ glucose utilization	No	[53]
	β-TCP, 14 mm diameter, 30 mm height	Human bmMSCs	Direct, 3–9 ml/min, 4 weeks	↑ differentiation, enter ↑ mineralization with increasing sheer; enter ↓ mineralization with increasing flow	No	[54]
Compression	Fibrin-demineralized bone, 15 mm diameter, 12 mm height	Human bmMSCs	4 kPa, 0.05 Hz, 24 h	↑ osteopontin, enter ↑ PDGF-α, ↑ TGF-β-R1	No	[58]
	β-TCP-MCPC, 6 mm diameter, 12 mm height	Human bmMSCs	5.5 ± 4.5 N, 0.1 Hz, 24 h	↑ Runx2; differential expression in 2 h	No	[59]
Compression and perfusion	Decellularized bovine bone, 20 mm diameter, 4 mm height	Human bmMSCs	10% compression, 0.5 Hz	↑ proliferation, enter ↑ osteocalcin	No	[60]
			10 ml/min, 3 weeks			
Hollow-fiber	Cytodex-collagen gel, >3.5 ml	Rat bmFCs	Cellulose acetate hollow fibers, 200 μm ID, 400 μm spacing	↑ proliferation, enter ↑ collagen remodeling and deposition	No	[63]
	Matrigel, >2 ml	Sheep bmMSCs	Polypropylene hollow fibers, 330 μm ID, 260 μm spacing	↑ proliferation, enter ↑ procollagen	No	[64]

PLGA, poly(lactic-co-glycolic acid); β-TCP, β-tricalcium phosphate; MCPC, monocalcium phosphate monohydrate; TMJ, temporomandibular joint; bm, bone marrow; MSCs, mesenchymal stem cells; FCs, fibroblastic cells; ALP, alkaline phosphatase; PDGF-α, platelet-derived growth factor-α; TGF-β-R1, transforming growth factor-β receptor 1

### Spinner-flask bioreactors

One of the simplest designs of an in vitro bioreactor is a spinner-flask. In this system, scaffolds seeded with cells are suspended from needles hung from the top of the bioreactor. Mixing of the media occurs via a stir bar or rod at the bottom of the vessel [37]. The convective forces assist with mass transport within the scaffolds hanging above, providing oxygen transport from the gas at the top of the vessel and removing waste products from within the scaffold. They can utilize the same cell culture media for the entirety of the experiment, exchange of the vessel media every 2 to 3 days, or continuous flow of media throughout the system. The flow generated within spinner-flask bioreactors tends to be turbulent and can vary with time [38].

Studies with spinning-flask bioreactors have shown promise for bone tissue engineering. Rat MSCs were seeded on porous cylindrical scaffolds comprised of poly(lactic-co-glycolic acid) (PLGA) (75:25, 81 kDa) that were 12.7 mm in diameter and 6 mm in height [39]. After seeding, the scaffolds were suspended from needles from the top of the vessel while the media was stirred at 30 rpm. At 7 and 14 days, the spinner flask vessels showed increased cellularity, and the cells within the scaffolds showed increased alkaline phosphatase expression (ALP) at day 7 and a higher calcium at day 14 when compared to scaffolds in static culture [39].

Likewise, larger porous silk scaffolds have also been used within spinning-flask bioreactors to successfully generate mineralized tissues. Human MSCs were cultured on aqueous-derived silk fibroin scaffolds of 15 mm in diameter and 5 mm in height for 84 days in both a spinning-flask bioreactor and static conditions [40]. The stir speed was 50 rpm. At early time points, scaffolds from the bioreactor had greater cell number and increased ALP activity. At later time points, bioreactor scaffolds had increased calcium deposition, modulus, and yield strength.

Many studies of small-volume scaffolds (<0.5 cm^3^) [38,41–43] show increased or similar proliferation and increased osteoblastic differentiation of the MSCs when compared to static culture. The use of larger scaffolds has demonstrated a nonhomogeneous distribution of cells, with increased cellularity at the edges of the scaffold [44]. It is possible that, while the spinner-flask bioreactor provides adequate nutrient exchange within the scaffold at earlier time points, at later time point—especially with deposition of ECM—the nutrient exchange is lacking within large scaffolds at later time points and may explain why other in vitro bioreactor types have more literature on larger scaffolds.

### Rotating-wall bioreactors

Rotating-wall bioreactors were originally designed to study the effects of microgravity on cells [45]. They consist of 2 concentric horizontal cylinders, with the outside cylinder rotating while the inside cylinder can be fixed or rotational. The cell aggregates or scaffolds are located between the 2 cylinders within culture media, while the gas exchange occurs via the inner cylinder. The flow within these systems is laminar, and shear stress can be reduced or controlled by varying the rotational rates of each of the cylinders [45]. By balancing the velocity of the fluid and the sedimentation rate of the scaffolds, the scaffolds remain in free-fall while still undergoing nutrient exchange.

Rotating-wall bioreactors are commonly utilized to culture cell aggregates or spheroids of several hundred micrometers [45]. Studies on larger clinically relevant scaffolds (>0.5 cm^3^) are lacking. One study utilized highly porous PLGA foams of 12.7 mm in diameter and 6 mm in length seeded with rat MSCs within a rotating wall bioreactor [44]. After 14 days in culture, the cell numbers were not significantly greater than static culture, but the distribution of cells was more uniform. There was no increase in osteocalcin over the static conditions, however. Another study using similar porous cylindrical PLGA foams, cells, and culture conditions showed more calcium deposition in the static culture conditions than in the rotating-wall bioreactors, despite the known benefits of forces on osteoblastic differentiation [39].

Scaffolds must remain in suspension within the rotating-wall bioreactor to prevent collisions with the inner or outer cylinder. The material density, as well as the porosity of the scaffold, media density and viscosity, and dimensions of the scaffold, plays an important role in the velocity of the scaffolds within the bioreactor [46]. As the tissue grows within the scaffold and alters the overall density, the speed of the rotating cylinders will need to be adjusted to keep the scaffolds in suspension [33]. Additionally, if culturing multiple scaffolds within the same reactor, scaffold aggregates can also form and affect cellular viability, both by increasing risks of collisions with sides of the vessel and limited transport within the center of the aggregate.

### Perfusion-based bioreactors

In perfusion-based bioreactors, the scaffold is placed directly within the vessel through which the media continuously flows. Direct perfusion bioreactors require a design such that the media flows directly through the scaffold; indirect perfusion bioreactors allow the media to flow around the scaffold, along the path of least resistance [47]. The direct bioreactors guarantee nutrient, gas, and waste exchange within the scaffold, but also expose the cells to higher shear forces, while the indirect bioreactors allow for nutrient exchange at the surface but rely on diffusion for the core of the scaffolds. Modeling the specific porosity of the scaffold along with the flow rate can allow for determination of the shear forces experienced by cells [48]. Perfusion bioreactors are the type of bioreactor most utilized for the generation of large clinically relevant mineralized tissues.

Anatomically correct structures have been generated within perfusion bioreactors. Using a computer-numerical-control milling machine and computed tomography (CT) images, a calf knee bone was shaped into a human mandibular condyle and seeded with human MSCs [49]. The initial seeding of both groups (direct perfusion or static) took place within a stirred tank bioreactor, and the cells were allowed to attach for 7 days before 4 weeks of additional culture. The perfusion group showed increases in cell number and mineralized tissue volume over the static group. Perfusion bioreactors have also been utilized to generate a segment of the largest bone in the human body—the head of the femur [50], raising the possibility that they could be used successfully for bones larger than a condyle, such as a whole mandible.

The media in indirect perfusion bioreactors follows the path of least resistance, which may be around the scaffold. Microporous β-TCP cylindrical scaffolds of 12 mm in diameter and 6 mm in height seeded with human MSCs were cultured in an indirect perfusion bioreactor [51]. While there was a significant increase in cell number in the perfused scaffolds relative to the static controls, there were no differences in the ALP per cell when the same serum-containing media was utilized. Further, the cells were well-distributed throughout the perfused scaffolds but remained superficial on the static scaffold.

Direct perfusion bioreactors encourage the perfusion throughout the whole scaffold, but the actual flow rates can vary significantly depending on the homogeneity of the porosity [49]. Human MSCs cultured on polyurethane foams showed more homogenous distribution throughout the scaffold than those cultured under static conditions; however, the ALP production per cell was similar [52]. Rat MSCs cultured on porous PLGA foams under direct perfusion also showed better uniformity throughout the scaffold than under static conditions [44]. The authors of this study showed higher ALP activity per cell in the perfused group.

Direct radial perfusion-based bioreactors have also been designed. Porous tubular scaffolds of β-TCP were created and seeded with human MG63 cells [53]. These were cultured under static conditions, with outward radial flow, or with inward media flow. The outward radial flow was shown to lead to the highest glucose consumption and cell number after 14 days. Another study on similarly designed porous tubular β-TCP scaffolds evaluated the effects of flow shear stress and mass transport on human MSC proliferation and differentiation [54]. By using different media flow rates and dextran concentrations within the media, the authors were able to create a variety of different flow shear stresses. The authors demonstrated that increasing flow shear stress increased differentiation and the formation of mineralized ECM, but that increasing mass transport impeded ECM mineralization.

Perfusion-based bioreactors allow for designs leveraging direct perfusion, indirect perfusion, or radial perfusion. Modeling of these systems is important as both shear stress and mass transport have been shown to affect MSC differentiation [55–57]. The porosity of the scaffold will change with time as the cells grow and deposit more ECM. Rather than maintaining a constant perfusion rate for the entirety of the experiment, the perfusion rate should be decreased in the direct and radial perfusion bioreactors [57]. In the indirect perfusion-based bioreactors, the inner core may become necrotic as the porosity of the exterior decreases and the media increasingly flows around the scaffold. When carefully designed, perfusion-based bioreactors can be utilized to generate mineralized constructs of clinically relevant sizes.

### Compression bioreactors

Compression bioreactors attempt to recapitulate the in vivo environment by applying stresses to the cells in an in vitro environment [58]. These systems can be designed to provide both static and dynamic forces. Compression bioreactors consist of a motor, a device providing linear motion, and a chamber with media in which the scaffold sits. Each episode of compression increases mass transfer (nutrient and oxygen delivery and waste removal) by circulating the surrounding cell culture media as well as applying compressive forces to the cells.

Applying compressive forces to MSCs has been shown to up-regulate genes associated with differentiation into osteoblasts after only 24 h of mechanical stimulation. Human MSCs were encapsulated in a fibrin-Dulbecco's modified eagle's medium matrix between 2 slices of rehydrated freeze-dried cancellous bone [58]. These scaffolds were then placed within a compression bioreactor that applied a pressure of 4 kPa with a frequency of 0.05 Hz, resulting in a deformation of about 1mm. The authors demonstrated that after 24 h in this environment the expression of several osteogenic genes was increased over the control (a scaffold placed within the same bioreactor without compression): Osteopontin saw a 2.6-fold increase in expression, while one of the receptors for TGF-β was increased 2.2-fold. Despite these promising results, the cells were not further studied to determine if in fact protein levels were increased or if these cells did in fact differentiate into osteoblasts while the control (no-compression) did not.

An additional study of clinically relevant sized constructs utilized porous ceramic scaffolds of 6 mm diameter and 12 mm length formed from β-TCP and monocalcium phosphate monohydrate [59]. In this study, human MSCs were seeded on one end of the cylindrical construct and allowed to adhere for 30 min before the other end of the construct was seeded. The ceramic scaffolds then underwent either diametral cyclic compression (5.5 ± 4.5 N at a frequency of 0.1 Hz) for 2 h or constant compression for 24 h. After 2 h of compression, there were differences between the static and the cyclic group. After resting for 22 h, Runx2 was significantly up-regulated in the cyclic group. Applying a constant compression for 24 h resulted in a >10-fold increase in gene expression of Osterix over the control.

Compression bioreactors can be combined with perfusion-based bioreactors. Human MSCs were seeded on bovine acellular matrix discs (20 mm diameter, 4 mm length) and subjected to a cyclic compression of 10% at a frequency of 0.5 Hz as well as an indirect perfusion flow rate of 10 ml/min [60]. The cell number and gene or protein expression were compared to static and perfusion-only conditions. After 3 weeks of culture, the cell number was significantly increased in the compression–perfusion and the perfusion-only bioreactors over the static controls. The amount of osteocalcin in the compression–perfusion was significantly increased over both the perfusion-only and the static culture conditions. While perfusion led to an increase in cell numbers, the mechanical stimulation was required to significantly influence cellular differentiation.

Compression-based bioreactors can be complicated to construct. In devices where one compressive head is to apply forces to multiple scaffolds, all scaffolds must be identical in height [33]. With increasing size of scaffolds, larger compression heads and motors are required, limiting intricate system designs to smaller tissue scaffolds or experiments that require testing of a single scaffold at a time. Additionally, while contamination of the cell culture media is a concern in any in vitro bioreactor that requires media changes, the additional complexity of compression bioreactors or combination compression–perfusion bioreactors can increase this risk [61].

### Hollow-fiber bioreactors

Another attempt to mimic the in vivo environment can be found with hollow-fiber reactors, which were designed to emulate native vasculature [62]. These bioreactors consist of a bundle of semipermeable hollow fibers through which oxygenated media is perfused. Cells within the surrounding hydrogel or within the adjacent scaffold can then exchange nutrients and waste products, similar to capillaries in the body. Cells adjacent to the hollow fibers are not exposed to any shear flow (as they are isolated from the flowing media), but the required number and spacing of these hollow fibers are governed by the diffusion through the external media or hydrogel.

A hollow-fiber bioreactor system was designed using cellulose acetate hollow fibers (inner diameter 200 μm, wall thickness 14 μm), with 200 hollow fibers at an approximate spacing of 400 μm [63]. Between the fibers, collagen gel containing microcarriers seeded with rat bone marrow fibroblastic cells was crosslinked. Cell media was then perfused through the hollow fibers at a rate of 14 ml/h (perfused) or just used to fill the fibers and exchanged daily (control). The authors found a significant increase in cell number in the perfused group. While the study did not look specifically at bone differentiation quantitatively, scanning electron microscopy of the perfused bioreactors showed cells migrating from the carriers and remodeling the collagen gel, laying down new collagen, while the nonperfused group showed cells remaining on the carriers and less new collagen deposition.

Another hollow-fiber system utilized sheep MSCs to look at the effects of microporous hollow fibers (330 μm inner diameter, 150 μm wall thickness) on proliferation and early bone differentiation [64]. The MSCs were then seeded between the fibers using chilled Matrigel, which polymerized upon raising the temperature of the system. The study investigated 2 different media flow rates (0.3 or 11 ml/min), corresponding to 2 different low and high Starling flows. The high Starling flow groups showed increased cell numbers, increased procollagen production, and collagen deposition by histological analysis.

Hollow-fiber bioreactors that allow for the generation of clinically relevant bone scaffolds mimic the Haversian canals found in native bone [65], with the media being perfused through the hollow fibers and the cells migrating between fibers. The design of the current systems requires that the MSCs be introduced in gel that can permeate between the fibers. This system may limit the ability to customize the scaffold porosity and properties; however, different crosslinking densities will affect mechanical properties of the gel and thus cell differentiation [66]. Additionally, small ceramic particles [16] or polymeric microparticles [67] can be introduced within a gel to encourage MSC differentiation.

## In Vivo Bioreactors

**Fig. 3.**
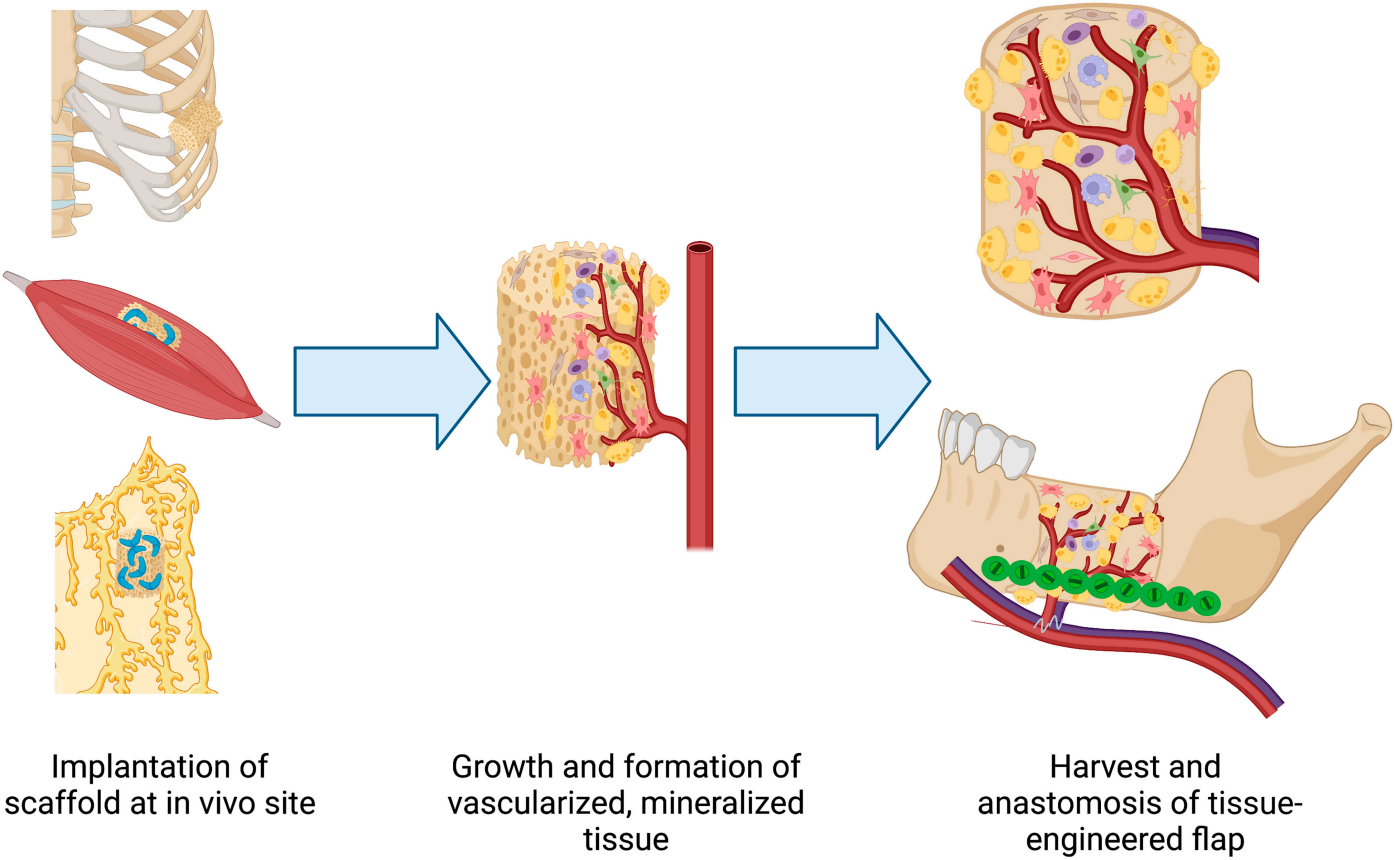
The strategy for in vivo bioreactor-based bone generation. In the first surgery, a scaffold is implanted adjacent to rib periosteum (no need for additional growth factors) or in a muscle or the omentum (likely requires addition of growth factors). Over the course of several weeks, cells migrate into the scaffold, and capillaries form to provide nutrients and gas exchange. The newly generated vascularized bone can be harvested with adjacent vasculature and transferred to the defect site.

As in vitro bioreactors try to recreate the oxygen delivery, waste removal, and mechanical cues that occur within the body, using the body itself as an “in vivo bioreactor” should also provide the necessary mass transfer and stimulus. Utilizing the in vivo bioreactor tissue generation strategy (Fig 3), a scaffold is implanted at a distal site away from the bone defect. Over time, mineralized tissue is generated within this scaffold. In a second procedure, the mineralized tissue is harvested and transferred to the site in need of repair. While there are several strategies for generating bone in situ [68], this section will focus on bone generation elsewhere in the body. Craniofacial defects that are infected or are created when cancer is resected cannot undergo immediate in situ repair due to biofilm formation on the implanted scaffolds or due to the stimulation of cancerous cells with growth factors, respectively. In these cases especially, it is advantageous to generate tissue elsewhere and transfer it after the defect site has been optimized. Bone tissue has been generated at several different sites in the body, with different scaffold materials, and with or without growth factors. A summary of these studies by site of implantation are given in Table 2.

**Table 2.** In vivo bioreactor studies.

Model	Location	Scaffold	Growth factor	Results	Source
Sheep	Rib periosteum	Autograft	No	Scaffold needed within bioreactors	[72]
		Autograft	No	9 weeks is optimum implantation time	[73]
		Autograft or MasterGraft	No	Bioreactor can be combined with space maintenance strategy	[74]
		Autograft or autoclaved Autograft	No	Loss of native growth factors caused decrease in mineralized tissue	[75]
		Autograft or MasterGraft	No	Bioreactor tissue can be successfully transferred as a flap	[76]
		Autograft or Bio-Oss	No	Distal processes can affect bioreactor bone generation	[77]
	Rib periosteum vs. muscle	Autograft	No	Periosteum resulted in more new bone formation than muscle	[82]
Pig	Muscle	Bio-Oss	BMP-7	Prefabrication better than in situ growth factor delivery	[83,84]
	Omentum	Calcium Phosphate	BMP-2	Significant vascularized bone formation at 8 weeks	[87,88]
Human	Rib periosteum	Autograft	No	Effectively augmented height of fibular flap	[79]
	Muscle	Bio-Oss	BMP-7	Improved mastication and aesthetics	[85,86]
	Omentum	Bio-Oss	BMP-2	Improved mastication, increased mineralization years after transfer	[89,90]

### Periosteal site

**Fig. 4.**
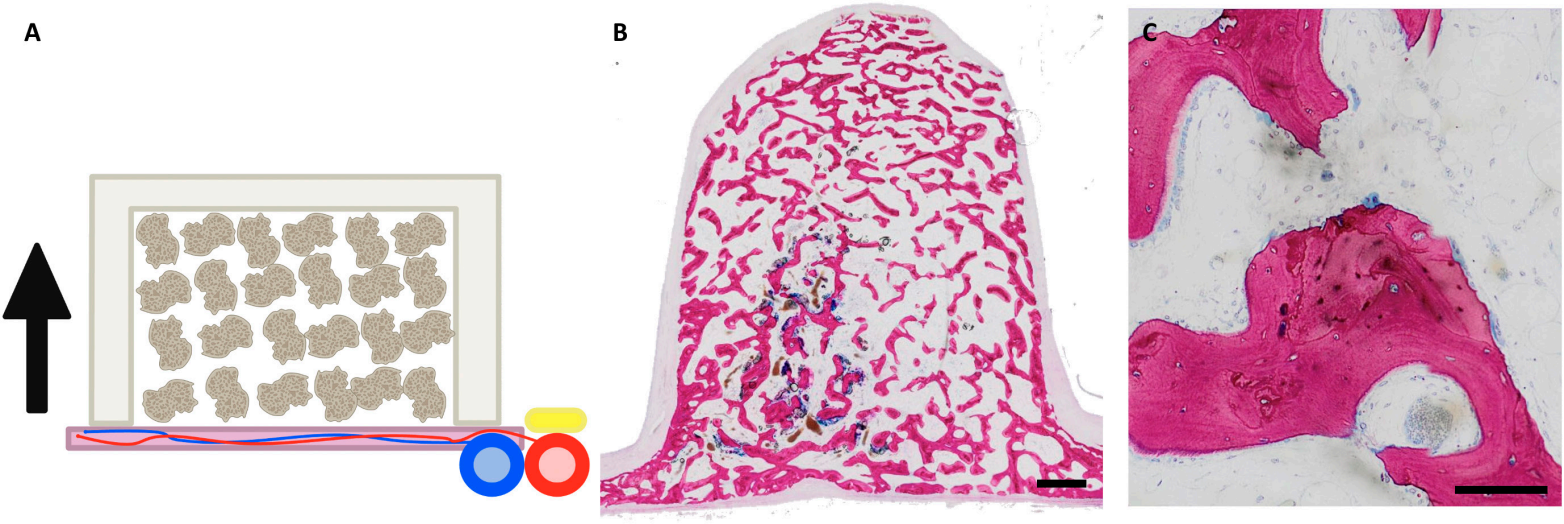
Histological images from an in vivo bioreactor implanted adjacent to periosteum of a sheep without the addition of growth factors (original images). (A) Schematic of bioreactor filled with scaffold (autograft, synthetic graft, or xenograft) adjacent to vascularized periosteum, with adjacent neurovascular bundle (nerve = yellow, artery = red, and vein = blue). Arrow indicating direction of bone growth. (B) Autograft-filled bioreactor after 9 weeks of implantation. More lamellar bone can be seen at the inferior edge (periosteal side) of the bioreactor, with some un-remodeled autograft remaining at the superior edge. Scale bar, 1 mm. (C) Osteoblasts can be seen lining newly deposited bone, with osteoclasts in resorption lacunae and viable osteocytes within the bone. An arteriole or venule filled with red blood cells can be seen among the fibrovascular tissue and adipocytes surrounding the bone. Scale bar, 100 μm.

The periosteum exists on every bone surface and consists of 2 layers: the fibrous and the cambium layer [69]. The cambium layer has a rich supply of pluripotent cells that can differentiate into osteoblasts, chondroblasts, or osteoprogenitor precursors. Additionally, macrophage-lineage tartrate-resistant acid phosphatase–positive cells can promote periosteal osteogenesis and regeneration by recruiting the pluripotent cells of the cambium layer [70]. The tissue is highly vascularized, and periosteal flaps have been shown to accelerate bone union and allograft vascularization [71]. The periosteum provides both a source of osteoprogenitor cells and growth factors and a rich capillary network for bone tissue engineering. The cells migrate up from the periosteum, generating scaffolds with more mature bone at the periosteal surface, and less mature bone further from the periosteum (Fig 4).

One of the earliest large animal studies of periosteal-based in vivo bioreactors was the sheep [72], which have ribs of similar size to humans. The bioreactors are created from poly(methyl methacrylate) (PMMA) that had been molded [73] or 3D printed [74] to the desired dimensions. The bioreactors are then filled with a morselized scaffold such as autograft [75], MasterGraft [76], or Bio-Oss [77] before implantation adjacent to the cambium layer of the periosteum. When the rib site is utilized, the intercostal artery and vein can be harvested alongside the tissue generated within the bioreactor, allowing for transfer of a vascularized flap [78]. This model has demonstrated the efficacy of the bioreactor-based strategy in generating mineralized tissues of up to 4 cm^3^ and—most importantly—that the generated tissue undergoes integration with the adjacent native bone after transfer and microvascular anastomosis [74]. The strategy requires no additional growth factors or cells, but the size of the generated mineralized tissue is limited by the surface area of the periosteum available.

The periosteum has been utilized in the clinic to create tissue to augment mandibular reconstruction [79]. The patient had undergone reconstruction with a fibular flap after resection of squamous cell carcinoma and radiation treatment; however, the fibula lacked the height necessary for the use of dental implants. A molded PMMA bioreactor filled with morselized autograft was implanted adjacent to the periosteum of the iliac crest for 8 weeks. The generated tissue was then harvested along with the periosteum and transferred to augment the mandibular height. After 1 year, histologic samples revealed adequately regenerated compact bone with numerous haversian systems and mature osteocytes, and the reconstructed mandible was capable of supporting dental implants.

### Muscle site

Muscle is highly vascularized and can be transferred as a flap along with engineered bone. Although it can provide blood supply and nutrient exchange, muscle lacks the osteoprogenitor cells and bone-specific growth factors found in periosteum [80]. For this reason, in vivo bioreactors implanted within muscle or adjacent to muscle fascia require the addition of growth factors (such as BMPs) or bone marrow MSCs [81]. Without any additional osteogenic cues, bioreactors implanted adjacent to muscle fascia grew entirely fibrovascular tissue and actually showed progressive resorption of the morselized graft [82].

The latissimus dorsi muscle of mini pigs has successfully been utilized as an implantation site for in vivo bioreactors [83,84]. Titanium cages were filled with Bio-Oss and loaded with BMP-7. After 6 weeks, the grafts were harvested with the adjacent thoracodorsal arteries. The tissue was transferred to a mandibular defect and anastomosed. The transferred tissue showed more robust repair than an identical contralateral defect treated with the same scaffold and growth factors in an in situ approach.

This strategy has also been utilized in humans [85,86]. Using computer-aided design, a titanium mesh was created based on patient imaging. The mesh was then filled with Bio-Oss and loaded with BMP-7 and bone marrow cell aspirate. After 7 weeks, the bioreactor was harvested and transferred to the mandibular defect and anastomosed. After 13 months, the patient still had a functional mandible, despite having infection of the hardware and cracking and exposure of the titanium mesh.

### Omental site

The omentum is another highly vascularized tissue that has been used to generate tissue-engineered bone flaps. Like muscle, an omental site also requires the use of BMPs or other osteogenic growth factors [87]. Bio-Oss blocks were soaked in BMP-2 and autogenous bone marrow aspirate before implantation within pouches close to vasculature within the gastric omentum [88]. After 16 weeks, the animals were euthanized, and the tissues were harvested for analysis. Bone was present within the scaffolds at the time of harvest, but transfer of the tissue to a defect site was not attempted.

A case study utilized the gastrocolic omentum to generate bone tissue for repair of a mandibular defect [89,90]. A titanium mesh filled with Bio-Oss and lined with a BMP-2-loaded collagen was implanted in a pouch at the distal end of the gastroepiploic arteries. After 3 months, the mandible was reconstructed with the generated tissues. Histological analysis demonstrated that the remodeling continued even after transfer of the tissue. This patient also experienced some complications secondary to the titanium mesh, so exploration into bioreactor materials that provide support to maintain scaffold shape and also degrade as bone growth occurs could yield improved results.

## Advantages and Disadvantages

In vitro and in vivo bioreactors both attempt to generate bone of clinically relevant sizes and geometries. Both strategies allow for scaffold customization—from size to porosity to material to overall geometry. Both strategies require at least one surgery and several weeks to months for generating the tissues. Although there are many similarities between the 2 strategies, different advantages and disadvantages exist between the strategies.

### Cell growth and differentiation

Both strategies rely heavily on the growth and plasticity of the MSCs from the patient. Aging [91], obesity [92], and health conditions like diabetes [93] have been shown to cause MSC senescence and affect their capacity for regeneration. In an in vitro bioreactor, additional growth factors or signals can be added to the media to encourage differentiation and deposition of mineralized ECM. However, in an in vivo environment, the tissue generation is entirely reliant on the host. The regenerative layer of the periosteum is at its thickest as a fetus and gradually thins with age [94] as the osteoprogenitor cells are lost. In vitro bioreactor strategies that utilize potent growth factors are less dependent on the innate regenerative capacity of the patient.

### Monitoring

As the generation of the mineralized tissue is very dependent on the patient, the time taken to adequately differentiate the cells and mineralize the scaffold will be somewhat variable person-to-person. In an in vitro bioreactor, the scaffold can easily be observed in a nondestructive manner. Substances in the media (glucose, lactate, etc.) can be measured as a surrogate for cellular activity, and CT of the scaffold can be utilized to follow mineralization [95]. In vivo, the bioreactor will be implanted beneath the skin, adjacent to a muscle or bone, or within the omentum of the abdominal cavity and cannot be easily observed. X-ray may be difficult to appreciate the mineralization within the bioreactor, and repeat CT testing could lead to excessive radiation exposure of the patient.

### Vascularization

In vitro and in vivo bioreactor strategies can both generate bone of >0.5 cm^3^. The in vitro strategy makes use of the flowing culture media for gas and nutrient exchange. In direct perfusion bioreactors, the media is forced to pass through the scaffold, with pores acting like a small capillary network. The initial cell population in the in vitro bioreactors is generally bone marrow MSCs, which can be forced to differentiate into endothelial cells in the presence of VEGF [96]. As this was not added in most studies, the finished mineralized tissue (while porous for media flow) will lack endothelial cells or capillary formation. Once implanted, the center of the mineralized tissue is reliant on convective flow from the extravascular fluid until capillaries can grow into the scaffold. In this case, it can take many years for the in vitro-generated autograft to incorporate completely [97].

The in vivo strategy is designed such that capillaries are generated alongside the bone tissue. As most of the scaffolds implanted are acellular, the osteoprogenitor cells and the endothelial cells will grow in at the same rate, forming vascularized bone. While initially reliant on the extravascular fluid to provide gas, nutrient, and waste exchange, the vessels will eventually form a lumen and mature, allowing for blood delivery to the site [98]. After the tissue has been generated, the vascularized tissue can be harvested with the adjacent blood supply. At the defect site, the pedicle of the tissue-engineered construct can be anastomosed to an established blood supply. The entirety of the tissue will receive gas and nutrient exchange via the blood supply immediately after transfer, decreasing risk of central necrosis.

### Translation

When developing strategies to be used in the clinic, it is important to consider the strategy for translation. The FDA regulates the commercialization of medical devices, biologics, and stem cells [99]. Devices that do not include biologics or stem cells and have a predicate device on the market have a much easier path to commercialization and can proceed along the 510(k) pathway [100]. Increasing complexity requires Pre-Market Approval (PMA) and large clinical trials. While an in vitro strategy for bone tissue engineering is not yet approved, Epicel is a strategy for burn wounds in which a postage-sized stamp of healthy skin is harvested, cultured, and expanded in vitro alongside mouse 3T3s, and then utilized as skin grafts for the same person [101]. The FDA granted this device a Humanitarian Device Exemption, which requires similar studies to the PMA but does not require proof of effectiveness, given the relatively few patients that would need it. The in vitro expansion must also be governed by good manufacturing practices. The use of growth factors within the bioreactors would also be required to proceed via the PMA pathway [102]. The product to be approved is the initial bioreactor implant. Depending on the complexity of the implanted bioreactor system, a simple scaffold created from material already approved in another device could require noticeably less paperwork and testing for FDA approval.

In addition to considering the commercialization strategy, it is important to consider the patient experience. The in vitro bioreactor strategy requires a cell harvest and a surgery for repair of the defect. The bone marrow can be aspirated from the iliac crest, and the procedure can be performed under regional anesthesia [103]. The most common adverse event is pain at the site. For the in vivo strategy, 2 separate surgeries must be performed, both requiring general anesthesia. The complexity of the surgery for the implantation of the bioreactor is highly dependent on the location the tissue is to be generated—a subcutaneous implantation is less involved than the removal of a rib segment to reveal the cambium layer of the periosteum. The repair surgery is also more involved, as the bioreactor tissue must be harvested along with adjacent vasculature, and then the vasculature must be anastomosed at the transfer site. The decision for which strategy to utilize should be a discussion between patient and physician considering all of the factors mentioned in this section.

## Conclusions and Future Directions

In summary, in vitro and in vivo bioreactors are a robust addition to the tissue engineering arsenal. They allow for the generation of mineralized tissues with increased cell numbers over similar scaffolds in static culture. In vitro bioreactors can also be utilized to increase homogeneity of cell seeding. Many in vitro studies existed for small scaffolds (<0.5 cm^3^), which provided important proof-of-concept information for the creation of larger scaffolds that could be useful for craniofacial defect repair discussed here. Despite progress over the years, neither in vitro nor in vivo bioreactors are perfect for every application, with in vitro eliminating many of the patient factors and allowing for close monitoring of the generated bone, while in vivo creates vascularized tissues that can be anastomosed to vasculature adjacent to the defect site. Both strategies will be complicated to commercialize and translate to clinics but can provide customized patient-specific strategies for high fidelity repair.

Future directions should involve leveraging the recent advances in bone tissue engineering with the optimized bioreactor settings discussed here. For scaffold generation, patient imaging (such as a CT scan) can be used to generate a computer-assisted design file that can then be utilized within a 3D printer to create a scaffold of exact dimensions and with specified porosity. Additional development of 3D printers has allowed for a wider variety of inks, such as the printing of decellularized ECM and calcium phosphate cements. 3D printing has also been utilized to allow for the printing of different materials or loaded with different growth factors, allowing for the creation of patterned scaffolds. Despite the design of such scaffolds and their success in tissue culture flasks, these complex scaffolds have yet to become widely utilized in bioreactors.

Another future direction to be explored is the utilization of cocultures on scaffolds in dynamic cultures. The in vivo strategy makes use of the many different cell populations present within the body; however, most of the in vitro strategies discussed utilize a single cell type. Although bone marrow MSCs can differentiate into osteoblastic, chondrogenic, or adipogenic cell lines, they cannot contribute the endothelial cells or monocyte derivatives (such as osteoclasts or macrophages) without the addition of a series of signaling molecules. Although cocultures of these cell types have been explored in direct or indirect under static conditions or as aggregates in dynamic cultures, studies of multiple cell types on scaffolds of clinically relevant sizes in dynamic cultures are lacking.

While cocultures could be utilized to generate vascularized bone, expansion of coculture to create additional adjacent tissue types could be advantageous for reconstruction. A mandibular condyle could be generated as discussed above, but to create a functioning temporomandibular joint, a cartilage surface would also be needed. In in vitro bioreactors, changing the mechanical properties of the scaffolds or the cells or growth factors incorporated within the scaffolds could assist with generating 2 different tissues in close proximity. Many of the flaps utilized in surgery can be harvested to include multiple tissue types (i.e., bone and skin, skin and adjacent muscle); however, additional research will be needed before in vitro bioreactors can generate such complex tissues. In vivo bioreactors could be placed such that adjacent muscle or skin could be harvested on the same pedicle to fully reconstruct the defect, although this strategy could add additional donor site morbidity.

Finally, many of the in vitro bioreactor studies studied the cells and their production of genes or proteins indicating osteoblastic differentiation and, in the longer studies, the mineralization of the final scaffold. However, these scaffolds should be transferred to an in vivo model after generation to truly determine the success of the strategy. While in vitro, the flow of the media ensures nutrient flow to the cells in the center of the scaffold; however, upon transfer to the in vivo defect, this media flow will no longer be present, and vasculature will need to be developed to provide oxygen and nutrients to these cells. To be a clinically relevant strategy, this transfer must be performed successfully. Ideally, the transfer would occur to a clinically relevant craniofacial defect; however, this is not often possible in the smaller proof-of-concept studies. Craniofacial bone tissue engineering—and the repair strategies available in clinic—will only continue to grow with further experiments utilizing in vitro and in vivo bioreactors, involving both the initial generation of bone tissue and reconstruction of the defect.
