# Corrigendum to “Advances in In Vitro and In Vivo Bioreactor-Based Bone Generation for Craniofacial Tissue Engineering”

**DOI:** 10.34133/bmef.0013

**Published:** 2023-03-27

**Authors:** Emma Watson, Antonios G. Mikos

**Affiliations:** Department of Bioengineering, Rice University, Houston, TX 77030, USA.

In the review article “Advances in In Vitro and In Vivo Bioreactor-Based Bone Generation for Craniofacial Tissue Engineering,” the authors made an error in Table. In Table, 10 cells in the Results column contain the word “enter,” which was erroneously added instead of starting a new line of text during proofing. This error did not affect the results, discussion, or conclusion of this paper. Table 1 has now been corrected in the PDF and HTML (full text).

**Table.** In vitro bioreactor studies that generate tissues of >0.5 cm^3^. Results relative to the control, most commonly static culture.

	**Scaffold**	**Cell type**	**Parameters**	**Results**	**Animal model**	**Source**
**Spinner-flask**	PLGA, 12.7 mm diameter, 6 mm height	Rat bmMSCs	30 rpm, 120 ml, 21 d	↑ ALP, ↑ osteocalcin, ↑ calcium	No	[39]
	Silk, 15 mm diameter, 5 mm height	Human bmMSCs	50 rpm, 100 ml, 84 d	↑ proliferation, ↑ differentiation early;↑ calcium, ↑ compressive modulus late	No	[40]
**Rotating-wall**	PLGA, 12.7 mm diameter, 6 mm height	Rat bmMSCs	6 foams, 20 rpm, 14 d	More homogeneous seeding, — proliferation	No	[44]
	PLGA, 12.7 mm diameter, 6 mm height	Rat bmMSCs	6 foams, 30 rpm, 21 d	— proliferation, ↓ calcium, ↓ALP	No	[39]
**Perfusion-based**	PLGA, 12.7 mm diameter, 6 mm height	Rat bmMSCs	Direct, 1.8 ml/min, 2 wk	↑ proliferation, ↑ ALP/cell	No	[44]
	Decellularized bovine bone, TMJ geometry	Human bmMSCs	Direct 1.8 ml/min, 4 wk	↑ proliferation, ↑ mineralization	No	[49]
	β-TCP, 12 mm diameter, 6 mm height	Human bmMSCs	Indirect, 1.5 ml/h, 3 wk	↑ proliferation, — ALP/cell	No	[51]
	Polyurethane, 24 mm diameter, 6 mm height	MC3T3-E1	Direct, 1.0 ml/min, 8 d	More homogeneous seeding, ↑ metabolic activity	No	[52]
	β-TCP, 14 mm diameter, 33 mm height	Human MG63	Direct, 3 ml/min, 4 wk	↑ proliferation, ↑ glucose utilization	No	[53]
	β-TCP, 14 mm diameter, 30 mm height	Human bmMSCs	Direct, 3–9 ml/min, 4 wk	↑ differentiation, ↑ mineralization with increasing sheer; ↓ mineralization with increasing flow	No	[54]
**Compression**	Fibrin-demineralized bone, 15 mm diameter, 12 mm height	Human bmMSCs	4 kPa, 0.05 Hz, 24 h	↑ osteopontin, ↑ PDGF-α, ↑ TGF-β-R1	No	[58]
	β-TCP-MCPC, 6 mm diameter, 12 mm height	Human bmMSCs	5.5 ± 4.5 N, 0.1 Hz, 24 h	↑ Runx2; differential expression in 2 h	No	[59]
**Compression and perfusion**	Decellularized bovine bone, 20 mm diameter, 4 mm height	Human bmMSCs	10% compression, 0.5 Hz 10 ml/min, 3 wk	↑ proliferation, ↑ osteocalcin	No	[60]
**Hollow-fiber**	Cytodex-collagen gel, >3.5 ml	Rat bmFCs	Cellulose acetate hollow fibers, 200 μm ID, 400 μm spacing	↑ proliferation, ↑ collagen remodeling and deposition	No	[63]
	Matrigel, >2 ml	Sheep bmMSCs	Polypropylene hollow fibers, 330 μm ID, 260 μm spacing	↑ proliferation, ↑ procollagen	No	[64]

PLGA, poly(lactic-co-glycolic acid); β-TCP, β-tricalcium phosphate; MCPC, monocalcium phosphate monohydrate; TMJ, temporomandibular joint; bm, bone marrow; MSCs, mesenchymal stem cells; FCs, fibroblastic cells; ALP, alkaline phosphatase; PDGF-α, platelet-derived growth factor-α; TGF-β-R1, transforming growth factor-β receptor 1.
